# Scalable fabrication of the graphitic substrates for graphene-enhanced Raman spectroscopy

**DOI:** 10.1038/s41598-017-09308-9

**Published:** 2017-08-17

**Authors:** Tommi Kaplas, Antti Matikainen, Tarmo Nuutinen, Sari Suvanto, Pasi Vahimaa, Yuri Svirko

**Affiliations:** 1 Institute of Photonics, University of Eastern Finland, FI-80101 Joensuu, Finland; 2 Department of Electronics and Nanoengineering, Aalto University, FI-00076 Aalto, Finland; 3 Department of Environmental and Biological Sciences, University of Eastern Finland, FI-80101 Joensuu, Finland; 4 Department of Chemistry, University of Eastern Finland, FI-80101 Joensuu, Finland

## Abstract

We propose direct synthesis of ultra-thin graphitic films on a dielectric substrate using sacrificial Ni catalyst layer, which significantly increases the crystallinity of the photoresist pyrolyzed at the temperature of 800 °C and above. A considerable amount of multilayer graphene in the photoresist film pyrolyzed in the presence of the Ni catalyst gives rise to an enhancement of the Raman signal of dye Sudan III molecules deposited on the substrate. We demonstrate comparable enhancement of the Raman signal from Sudan III molecules deposited on the fabricated graphitic substrate and those deposited on graphene, which was conventionally transferred to the silica substrate.

## Introduction

Ever since graphene isolation 15 years ago, a variety of applications have been proposed and demonstrated for both single and multilayered graphene^1–3^. However, the direct synthesis of these highly ordered atomically thin graphitic films on dielectric and semiconductor substrates remains a difficult task. This is partially because the chemical vapor deposition (CVD), a conventional graphene synthesis technique, relies on a metal (e.g. Cu and Ni) catalyst^3^, while applications usually require graphene deposited on a dielectric/semiconductor substrate. The transfer of an atomically thin film essentially involves manual processing, which is the major bottleneck in the implementation of graphene in practical devices. The direct and scalable graphene synthesis on insulating substrates is an eagerly awaiting breakthrough that should open pathways towards an industrial scale exploitation of graphene in sensing, electronics and photonics^4^.

In the search for the methods of the direct graphene deposition there have been proposed CVD based techniques that exploit sacrificial ultrathin copper or nickel catalyst films pre-deposited on the target dielectric substrate rather than bulky metal foils^5–8^. In the framework of such an approach, one needs just to remove the remains of the metal film from the substrate in order to obtain graphene deposited on the dielectric substrate. Although this technique is relatively simple and straightforward, the electronic properties of the obtained polycrystalline single- and multilayer graphene are usually poorer than those of the transferred one^5^. Nevertheless directly deposited graphitic films are suitable for a number of applications in optics and photonics^4^.

Graphene enhanced Raman spectroscopy (GERS) manifests itself as the enhancement of the Raman signal from an analyte deposited onto the graphene sheet. In contrast to the conventional surface-enhanced Raman spectroscopy (SERS), which exploits local field enhancement in the vicinity of metal nanoparticles deposited on the dielectric substrate, GERS is driven by the charge transfer between the analyte and graphene^9^. This enhancement mechanism is well balanced and delicate system affected by the graphene Fermi level, highest occupied molecular orbital (HOMO) and lowest unoccupied molecular orbital (LUMO) energy levels of the analyte, excitation laser wavelength and the structure of the analyte molecule^10^. Together with ability of graphene to protect the analyte from photobleaching and suppress fluorescence such a specificity may be highly beneficial for molecular spectroscopy^11^.

In this paper, we demonstrate wafer scale direct deposition of multilayer graphene on a dielectric substrate by using pyrolysis of photoresist film, which is graphitized at 800 °C in presence of a 10 nm thick nickel nanocatalyst layer. We show that fabricated films can be used for GERS of Sudan III dye molecules, an illicit, carcinogenic food and fuel marker dye^12^. We demonstrate in particular that fabricated GERS-active substrates provide the enhancement of the Sudan III Raman lines comparable to that can be obtained by using graphene conventionally grown on a copper foil and transferred onto a dielectric substrate.

## Results and Discussion

**Figure 1:**
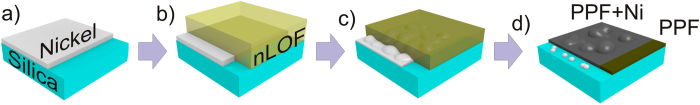
Sketch of the direct synthesis of the graphitic film on a dielectric substrate. (**a**) Physical deposition of the 10 nm thick Ni film; (**b**) Spin coating of the Ni with nLOF resist layer. (**c**) The pyrolysis of the resist and morphological changes in the Ni layer start at 600 °C; (**d**) At 800 °C the resist film is pyrolyzed and Ni film receds to nanoparticles.

**Figure 2:**
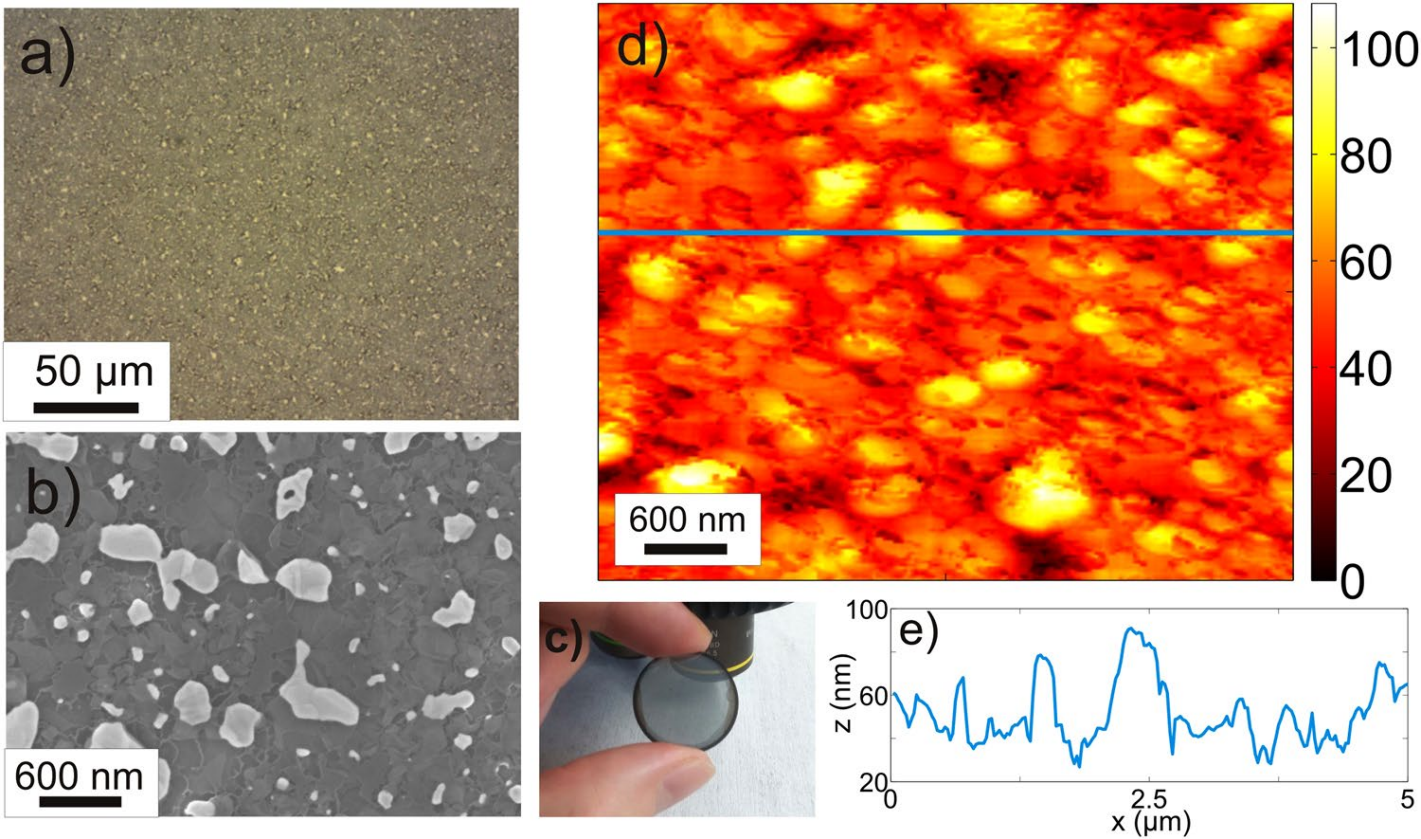
Optical microscope (**a**) and scanning electron microscope (SEM) (**b**) images of the PPF + Ni film show the Ni particles on a sample surface. (**c**) Photographic image of the semitransparent PPF + Ni. (**d**) An atomic force microscopic image shows that the height of the largest Ni particles is around 100 nm. (**e**) The film thickness plotted along the blue line shown in (**d**).

In order to fabricate the graphitic film, we evaporated a 10 nm (±2 nm) thick nickel catalyst layer on a 0.5 mm thick silica substrate, spin coated it with nLOF resist (by Microchemicals ltd), and then baked the structure in hydrogen atmosphere (with 5 sccm flow, 0.5 mBar pressure) at temperature 600 °C–900 °C. Schematic illustration of the graphitic film preparation is shown in Fig. 1. Although, the melting temperature of bulk nickel is 1455 °C, we observed change of the Ni film morphology at temperature of as low as 700 °C. Specifically, between 700 and 800 °C, the pyrolyzed photoresist film (PPF) synthesized in presence of the Ni film (will be referred to as PPF + Ni film) underwent remarkable transformation and at the process temperature of 800 °C, the melted Ni film receded to an array of micron- and submicron sized nickel particles (See Fig. 2), which remained nearly unchanged when the process temperature was increased up to 900 °C.

The graphitic film surface morphology and thickness were studied by atomic force microscope (AFM - Thermo Microscopes Explorer 4400–11) and thickness by stylus profiler (Weeko Dektak 150), respectively. Figure 2 shows that the height of the largest Ni particles is order of hundred nanometers, while their lateral size is in sub-micron scale. The smallest Ni particles have nearly spherical shape and lateral size of a few tens of nanometers. The Ni particles are embedded into the 20–25 nm thick carbon film, which is about 50 times thicker in comparison to single graphene layer. The surface roughness of the PPF + Ni film averaged over the sample area is as high as 50 ± 0.5 nm, while that of PPF film pyrolyzed without Ni is 6.4 ± 1.0 nm (see also Supplementary information).

To reveal how the presence of the Ni layer affects the graphitization, we measured the Raman spectra of the fabricated samples using 514 nm excitation wavelength. In order to avoid effects of the graphene self-heating the excitation beam power was fixed at 0.25 mW^13^.

**Figure 3:**
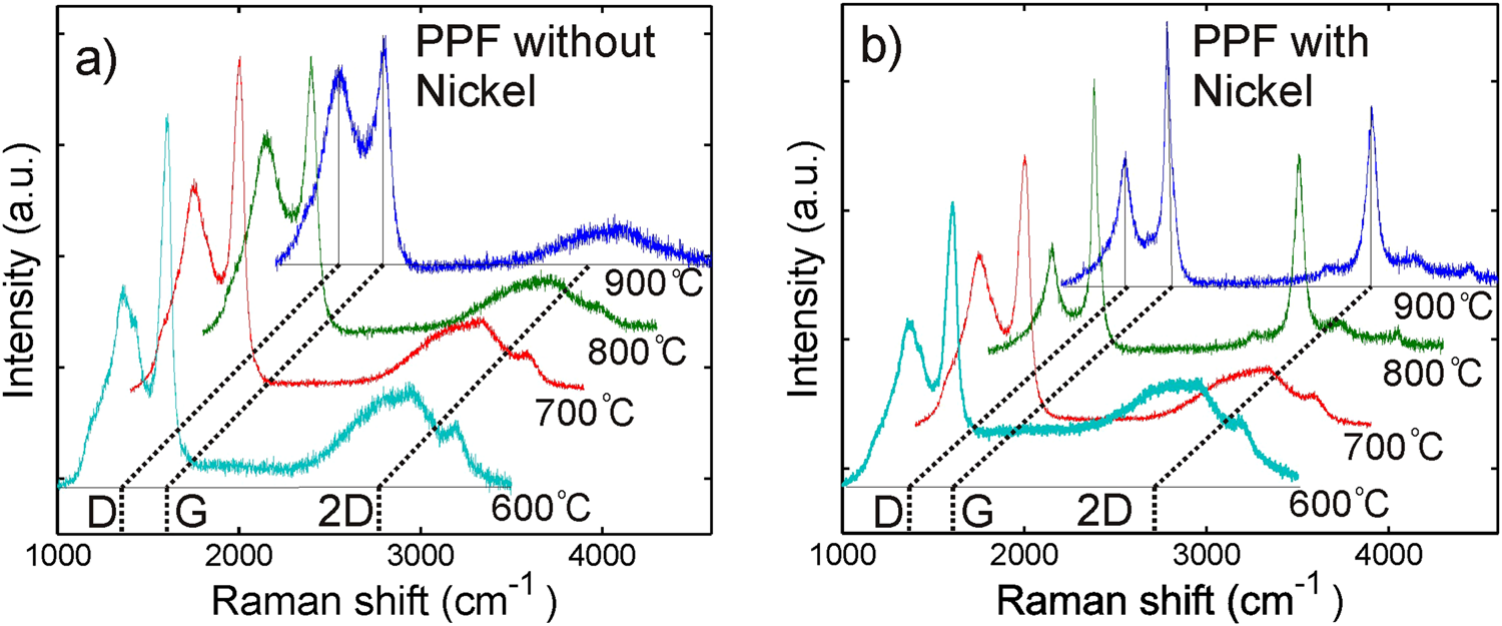
Raman spectra of carbon films fabricated at different temperatures with and without a Ni film. (**a**) Raman spectra of PPF correspond to very amorphous carbon material and show minor changes temperature is increased. (**b**) Raman spectrum PPF+Ni changes drastically between 700 °C and 800 °C.

The Raman spectra of samples processed at temperatures of 600 °C and 700 °C shown in Fig. 3 are featured with strong and wide D- and G-peaks (~1350 cm^−1^ and ~1580 cm^−1^, respectively)^14^. The 2D peak at ~2700 cm^−1^, a widely recognized signature of the crystalline graphene^14, 15^, is barely visible for the films pyrolyzed both with and without Ni catalyst.

When the process temperature exceeds 800 °C the Raman spectrum of the PPF remains almost unchanged, while the Raman spectrum of the PPF+Ni changes drastically. Figure 3 shows that in the Raman spectra of the PPF+Ni processed at 800 °C and 900 °C, D- and G- peaks become sharper and the 2D peak becomes clearly observable being comparable to the D- and G-peaks. These observations indicate that in the film pyrolized at temperature higher than 800 °C, the amount of amorphous carbon decreases, while the size of graphitic, sp^2^ hybridized domains increases^14^.

Next, we performed GERS experiments with films fabricated at different process temperatures using aromatic azo dye (Sudan III) as a target analyte (see the Supplementary information for the Raman bands of Sudan III and further experimental GERS details). In the experiment, droplets of 0.1 mM Sudan III diluted in acetone were placed on the surface of the PPF and PPF+Ni samples and dried out.

**Figure 4:**
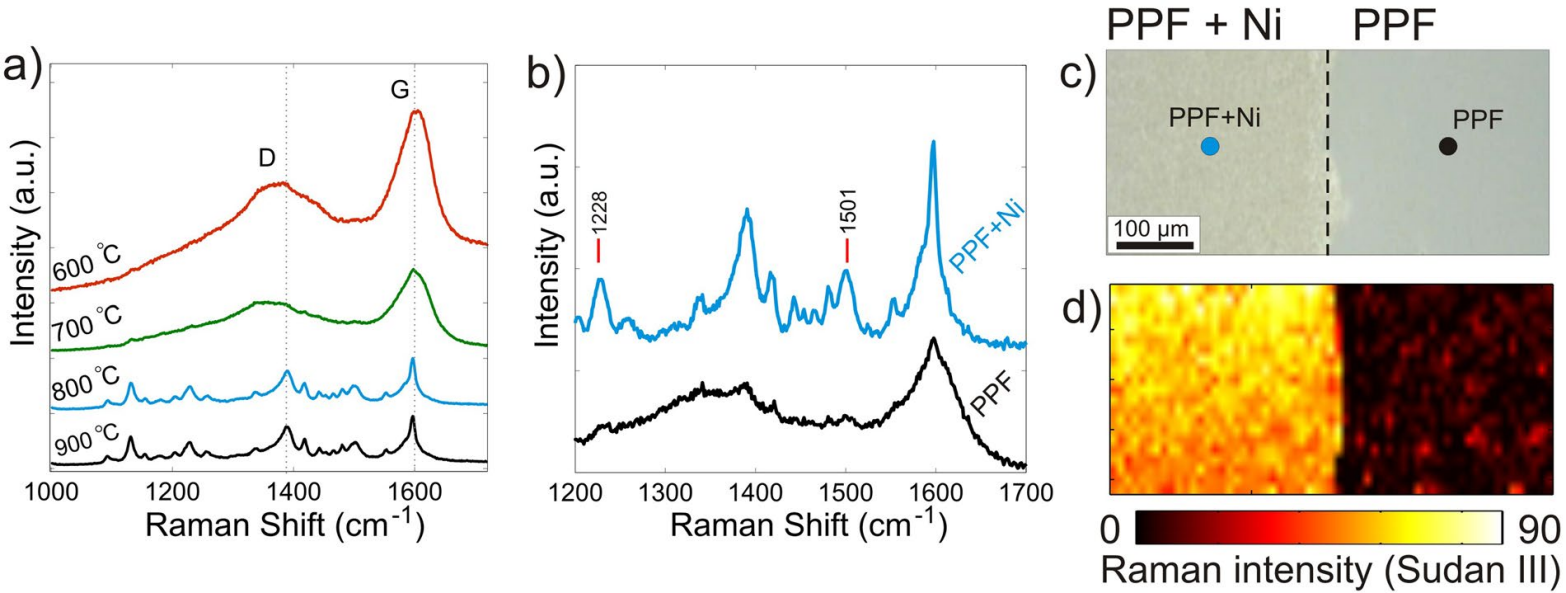
GERS spectra of Sudan III molecule. (**a**) The PPF+Ni process temperature dependence to the resulting GERS enhancement shows remarkable difference between sample fabricated at 700 °C and 800 °C. (**b**) Raman spectra for the PPF+Ni and PPF fabricated at 800 °C (averaged over the substrate surface). (**c**) Optical microscope image showing the boundary between PPF+Ni and PPF. (**d**) Mapping of the Sudan III Raman peak at 1228 cm^−1^ at the boundary between PPF+Ni and PPF substrates. In both (**a** and **b**) the spectra have been displaced for clarity.

Measured Raman spectra of the prepared samples are presented in Fig. 4. The Raman spectra of the samples prepared with graphitic films deposited at temperatures 600 °C and 700 °C show no peaks from Sudan III (Fig. 4a). However, in the Raman spectra measured by using PPF+Ni substrates fabricated at higher temperatures, the Raman peaks from the Sudan III become clearly visible. Thus, Raman scattering cross section of Sudan III molecules is enhanced when they are deposited on the carbon film with enhanced crystallinity.

In order to visualize the effect of the Ni catalyst on GERS, we performed mapping of the Raman signal at the boundary between PPFs synthetized with and without the sacrificial Ni layer (Fig. 4c and d, respectively). The mapping reveals a drastic difference between GERS performance of these substrates. Specifically, the Raman peaks from Sudan III are clearly visible when it is deposited onto PPF+Ni, while they are barely distinguishable from the noise when deposited on the bare PPF. By comparing the strongest peaks at 1228 and 1501 cm^−1^ (see Fig. 4b) one may conclude that the Raman signal from the Sudan III molecules deposited on the PPF+Ni is about one order in magnitude higher than that from the Sudan III molecules deposited on the bare PPF.

To visualize the contribution of Ni particles to the Raman enhancement, we removed Ni with wet etching solution (Supplementary information), rinsed the sample in water and compared Raman spectra from the PPF with and without nickel particles. One can see from Supplementary information Fig. S3 that removing the Ni particles does not decrease the magnitude of the Raman signal of the Sudan III. Furthermore, we observed similar enhancement of the Raman peaks of the Sudan III deposited on the graphene, which was fabricated on copper foil and transferred on a silica substrate^16^. These experimental findings clearly indicate that enhancement of the Raman signal originates from the presence of the graphene flakes on the silica substrate.

Typically, CVD graphene is formed in the temperature range 900–1000 °C^5, 6, 17^, i.e. it takes place well above melting temperature of inexpensive and flexible glass substrates^18^. In contrast, the PPF+Ni with improved crystallinity can be fabricated at a considerably lower temperature, which can open a way for a scalable fabrication of the GERS substrates by using inexpensive boron silica glass, which is widely used in chemical industry.

We believe that one major reason for the formation of the graphitic carbon film is a high carbon solubility in nickel. One may expect that above 700 °C, the carbon solubility increases drastically giving rise to merging Ni and the resist films^19^. In this temperature range, Ni catalyzes transformation of the amorphous carbon into graphitic crystal while the Ni film will melt and recede. This process resembles multilayer graphene synthesis on the surface of the Ni foil, when dissolved carbon atoms leaves the foil as soon as the temperature in the CVD chamber starts decreasing^20^. In our experiment situation is similar - Ni particles in the resist film recede leaving graphitic crystal behind.

Since melting and receding of Ni film are very random processes, one may expected that the graphitic film, which is formed between Ni particles, is rather defected. The relative intensities of the D- and G-peaks in the Raman spectrum (see Fig. 3) indicate that size of graphene crystallites in the fabricated films is rather limited^14, 15, 21^. However, the strong 2D-peak, suppressed D-peak and sharp G-peak indicate that the adding Ni catalyst results in higher degree of crystallization in comparison to the PPF on a bare silica substrate^14, 15, 21^. That is despite the fact that PPF+Ni is multilayered and defected film, it performs well in terms of GERS. This experimental finding well corresponds to earlier observations that multilayer graphene can have as good GERS properties as monolayer graphene and that defects can even improve the GERS signal^22, 23^. Therefore, the proposed nickel nano-catalyst assisted growth of multilayer graphene can be employed for scalable fabrication GERS substrates capable of provide an increase of the Raman signal comparable to that of conventional transferred graphene. Moreover, the proposed technique allows one to either remove Ni particles if required but otherwise can provide a nice way to obtain metal-graphene composite material, which may lead into useful applications^24, 25^.

Overall, GERS is a highly selective process and it has been demonstrated that the GERS effect may occur when one of two molecular selectivity rules is satisfied. (i) The energy rule, which states that in order to generate significant enhancement, HOMO/LUMO energy levels of the analyte molecule need to be close enough to the Fermi level of graphene^26^. (ii) The structure rule, according to which the GERS enhancement can occur if the analyte molecule is structurally “compatible” with graphene. For example, it may have D_nh_ symmetry or it may consist of parallel connected conjugated benzene rings, which match the structure of graphene enabling strong coupling (and charge transfer)^26^.

The HOMO/LUMO energy levels of Sudan III are −5.8 eV and −3.1 eV, respectively^27^, while the Fermi energy level of the graphene and graphite is at −4.6 eV. Therefore, for the 514 nm laser, the energy level rule does not give a justification for GERS^26^. Thus, we believe that in case of Sudan III, the enhancement is related to the structure rule rather than the energy rule. Although, Sudan III is non-symmetrical molecule, it has a parallel conjugated carbon ring structure that enables coupling to the graphitic PPF+Ni substrate, which in turn increases π-π interaction and thus enhances the Raman signal^10, 26^. On the contrary, benzene rings of Sudan III cannot couple to the sp^2^ orbitals of strongly amorphous PPF implying that no enhancement can take place.

It is also worth noting that the Raman enhancement is slightly stronger in PPF+Ni in comparison to a monolayer graphene fabricated by a conventional transfer technique (see Supplementary information). Here the difference between PPF+Ni and transferred graphene most likely originates from the rough surface of the PPF+Ni sample, as the roughness increases the surface area, which can then consequently increase the number of analyte molecules coupled to the PPF+Ni substrate surface. Thus, the presented technique, which results in rougher graphitic films, can actually be beneficial to GERS.

Finally, in order to confirm that presence of the graphene flakes on the rough surface of the PPF+Ni film enhances Raman scattering of Sudan III, we fabricated also a rough PPF described in Supplementary information. However, no enhancement of the Raman signal was observed when Sudan III molecules were deposited on such a rough PPF substrate. This experimental finding clearly indicates that the enhancement of the Raman signal with PPF+Ni substrate, indeed, originates from the GERS rather than simply the increase of the surface area.

## Conclusion

In summary, we have proposed a scalable and inexpensive technique for a GERS substrate fabrication by pyrolysis of the photoresist film deposited on a silica surface coated with a 10 nm thick Ni layer. We observed that at temperature of about 800 °C the Ni film melts and recedes to submicron size Ni particles. At this same temperature range the catalysis of Ni begins and the amorphous PPF undergoes transformation from amorphous carbon to polycrystalline graphitic film. This transformation was observed with Raman measurements. Furthermore, the Raman enhancement of Sudan III molecule on this graphitic film was observed to be about one order of magnitude. It is well-known that GERS increases the Raman signal much less in comparison to those metallic SERS platforms^26, 28^. However, rather high surface roughness of the fabricated PPF+Ni film improved slightly Raman scattering in comparison to the transferred, flat graphene substrate.

## Materials and Methods

### Sample fabrication

Nickel thin film was evaporated on a 0.5 mm thick, 25 mm in diameter fused silica substrate. Thermal evaporation was done by Leybold Univex 300 in 10^−5^ mBar vacuum using 99,99% pure Ni pellets. Sample was next coated with a resist (AZ 2070 nLOF by Microchemicals diluted with AZ EBR with 1:4 ratio, respectively) spin coating at 2000 rpms. Spin coating resulted in about 230 nm thick resist film on the Ni+silica substrate.

Samples were pyrolyzed in a chemical vapor deposition (CVD) apparatus. The CVD chamber was pumped for one hour in 5 sccm H_2_ flow in order to remove oxygen from the chamber. Next the chamber temperature was risen to the actual process temperature by 20 °C/min. After 10 min baking in the chosen process temperature the sample was cooled down to room temperature (overnight) in static 8 mBar H_2_ atmosphere.

### GERS experiment

GERS measurements were performed using Renishaw inVia Raman microscope with 514 nm excitation and Sudan III as the target molecule. The samples were prepared for the measurements by depositing a 2 µl droplet of 0.1 mM Sudan III (analytical grade ≥96%, Sigma-Aldrich) diluted in acetone on the surface of the samples. The temperature dependence measurements were carried out using 0.25 mW power, 10 s integration time and 100X objective. In total, 9 measurements were recorded for each sample (600 °C, 700 °C, 800 °C and 900 °C) and the spectra were then averaged. The Raman mapping measurements were collected from a 200 µm × 600 µm area at 10 µm intervals using 0.5 mW power, 5 s integration time and 20X objective.

## Electronic supplementary material

Supplementary information
