# Supplementary material for: Scalable fabrication of the graphitic substrates for graphene-enhanced Raman spectroscopy
Source: Sci Rep. 2017 Aug 17;7:8561. doi: 10.1038/s41598-017-09308-9 (PMC5561221; doi:10.1038/s41598-017-09308-9)
Supplement: Supplementary file 1 — Supplementary information [file 41598_2017_9308_MOESM1_ESM.pdf]

Supporting information

Scalable fabrication of the graphitic substrates for graphene-enhanced Raman spectroscopy

Tommi Kaplas,[1,*] Antti Matikainen,[1,2] Tarmo Nuutinen,[1,3] Sari Suvanto,[4] Pasi Vahimaa,[1] and Yuri Svirko[1]

[1]*Institute of Photonics, University of Eastern Finland, FI-80101 Joensuu, Finland*

[2]*Department of Electronics and Nanoengineering, Aalto University, FI-00076 Aalto, Finland*

[3]*Department of Environmental and Biological Sciences, University of Eastern Finland, FI-80101 Joensuu, Finland*

[4]*Department of Chemistry, University of Eastern Finland, FI-80101 Joensuu, Finland*

*Corresponding author: tommi.kaplas@uef.fi

Surface roughness

Surface roughness was measured with and without Ni nanocatalyst by using AFM - Thermo Microscopes Explorer 4400-11. Results are shown in Fig S1.

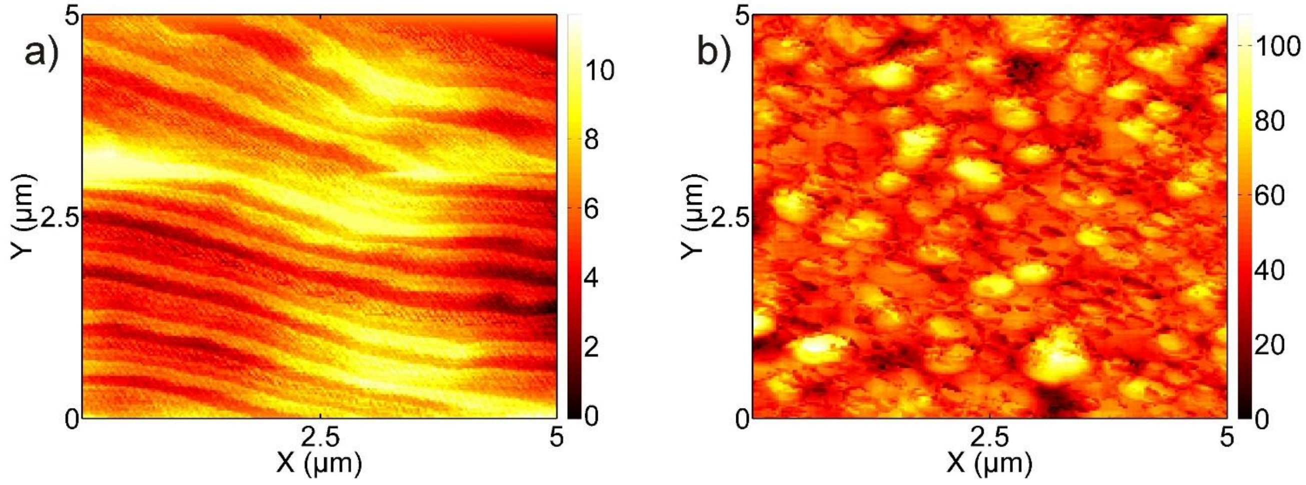

Figure S1. AFM images of the PPF surface with and without Ni nanocatalyst layer. (a) Without Ni, the PPF has very smooth surface. The surface seem to have periodical waves with height of about 5 nm. These waves are supposed to originate from the spin coating of the resist film. (b) When Ni catalyst film melts and recedes, the surface roughness increases by one order of magnitude. Randomly placed Ni particles are seen rather clearly in AFM image.

Average surface roughness (R_a) was calculated by Eq (1):

$$R_a = \frac{1}{L}\sum_{k=0}^{L}|x_k|, \qquad (1)$$

where L is measured length, k label the data points from 0 to L, and x_k is the surface height measured in *k-th* point

Raman peaks of Sudan III

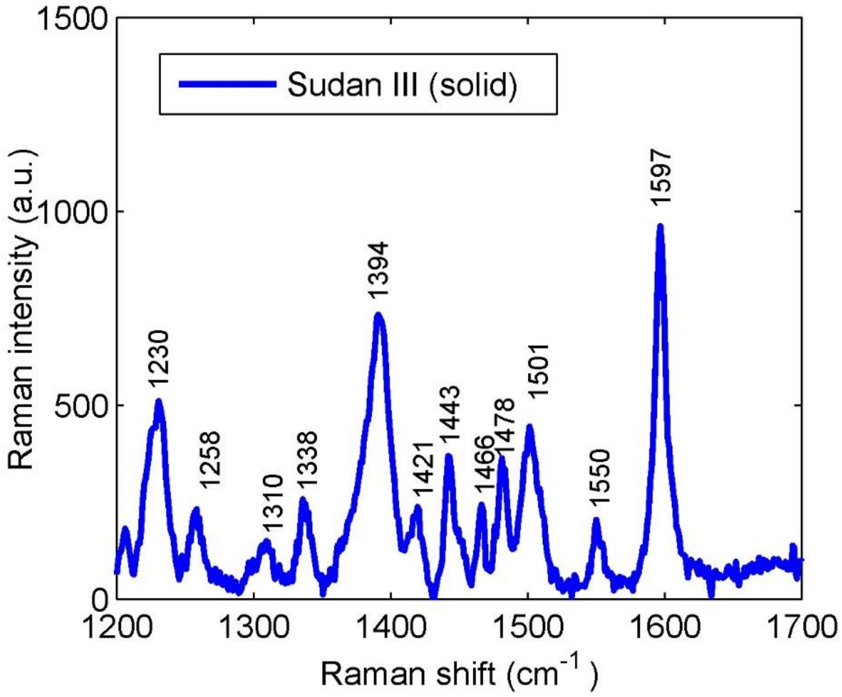

Figure S2. Raman peaks of a solid Sudan III sample.

Reference measurement with transferred graphene

Although there is a common belief that Ni is an SERS-inactive material, it is reasonable to assume that remaining nickel particles are still capable to give a finite contribution to the observed Raman enhancement. This contribution was further examined by (i) removing remained nickel by wet etching ($CuSO_4$-HCl-H_2O solution) and (ii) measuring GERS on a graphene sample prepared by conventional transfer technique. Since Raman enhancement from Sudan III target molecule in (i) and (ii) was comparable to that of obtained with PPF with Ni particles, we may exclude the effect of the local field enhancement at Ni particles in this experiment.

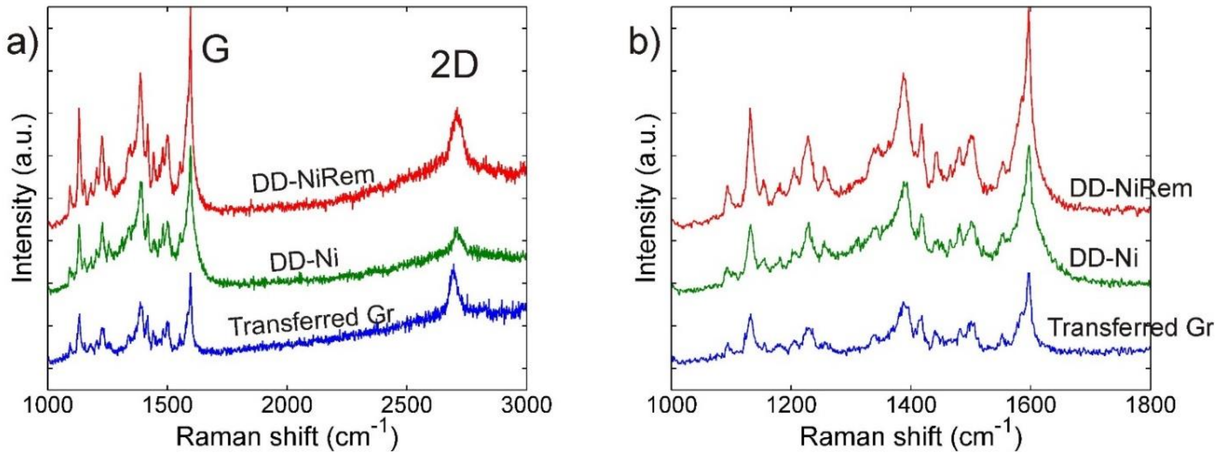

Figure S3. Raman enhancement on transferred graphene (Transferred Gr), directly deposited graphene with Ni particles (DD-Ni) and when Ni is removed (DD-NiRem). (a) Typical peaks for graphite, G and 2D peaks can be seen on all samples. However, it is noteworthy that the 2D peak position of the transferred graphene is located at around ~2685 cm-1, while it is around ~2710 cm-1 for directly deposited multilayer graphite. (b) The magnitude of Sudan III peaks are comparable in all of the samples.

Rough amorphous PPF reference

A rough, amorphous carbon reference sample was fabricated to demonstrate that surface roughness of the PPF does not increase the Raman scattering but the enhancement origins from the chemical mechanism.

To make the sample we evaporated 10 nm of gold and nickel on a silica substrate so that the thickness of the combined metal film was 20 nm. Next the metal film was coated with nLOF resist and the sample was baked at 900 C. In the process Au merges to Ni and passivates the Ni catalyst. The resulted amorphous carbon film consist of an amorphous carbon film with Ni-Au particles with approximately the same size in comparison to the Ni-PPF sample.

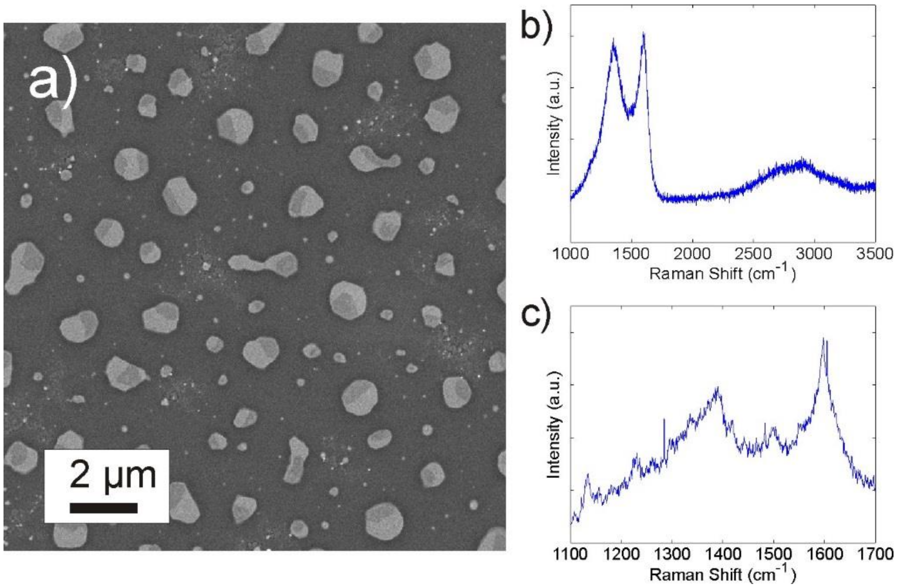

Figure S4. (a) A SEM image of an amorphous carbon film with sub-micron Au-Ni-metal particles. (b) The Raman spectrum from the Au-Ni-PPF (without Sudan III) shows only amorphous trace of carbon. (c) When Sudan III analyte is deposited on the Au-Ni-PPF surface, the Raman signal is not enhanced.
